# Co-Occurring Genomic Alterations in NSCLC: Making Order into a Crowded List

**DOI:** 10.3390/cancers17142388

**Published:** 2025-07-18

**Authors:** Ilaria Attili, Federico Pio Fabrizio, Filippo de Marinis

**Affiliations:** 1 Division of Thoracic Oncology, European Institute of Oncology, IEO, IRCCS, 20141 Milan, Italy; ilaria.attili@ieo.it (I.A.); filippo.demarinis@ieo.it (F.d.M.); 2 Department of Medicine and Surgery, University of Enna “Kore”, 94100 Enna, Italy

**Keywords:** non-small cell lung cancer, co-occurring genomic alterations, molecular profiling, tyrosine kinase inhibitors, targeted therapies

## Abstract

Worldwide, lung cancer is one of the most common cancers, with non-small cell lung cancer (NSCLC) including up to 80–85% of all lung cancer diagnoses. The landscape of NSCLC is characterized by a heterogeneous spectrum of gene alterations, with tyrosine kinase inhibitors (TKIs) and targeted treatments that significantly improve survival outcomes for patients with oncogene-addicted NSCLC, offering superior efficacy, and often favorable safety and tolerability profiles compared to chemotherapy-based treatments. However, the complexity of NSCLC extends to co-occurring genomic alterations or amplifications in tumor suppressors and other oncogenes, such as *TP53*, *STK11*, *KEAP1*, *PIK3CA*, *RB1*, and others, that significantly influence disease progression, therapeutic resistance, and clinical outcomes. These co-mutations often contribute to the development of primary and acquired resistance to targeted therapies, complicating decision-making strategies. This review provides a timely and comprehensive synthesis of current insights into co-mutations in NSCLC, with a particular focus on their clinical implications, and offers a novel perspective by integrating recent molecular insights with therapeutic challenges, addressing existing knowledge gaps through a more integrative and clinically oriented analysis of co-mutations. Advances in next-generation sequencing (NGS) and molecular profiling have enabled the identification of these co-alterations, paving the way for more personalized therapeutic approaches. However, challenges remain in interpreting the functional interplay of co-mutations and translating these insights into effective clinical interventions. This review also highlights the significance of co-mutations in shaping NSCLC biology, and discusses their impact on current therapeutic paradigms, emphasizing the need for integrative biomarker-driven approaches to improve outcomes in NSCLC.

## Simple Summary

Non-small cell lung cancer (NSCLC) often harbors oncogenic driver alterations such as epidermal growth factor receptor (EGFR), anaplastic lymphoma kinase (ALK), or Kirsten rat sarcoma viral oncogene homolog (KRAS), which can be effectively targeted with tyrosine kinase inhibitors (TKIs). However, tumors frequently contain additional co-occurring mutations in genes like tumor protein p53 (TP53), serine/threonine kinase 11 (STK11), and Kelch-like ECH-associated protein 1 (KEAP1), which may affect tumor growth, immune evasion, and treatment response. These co-mutations contribute to molecular heterogeneity and are associated with worse prognosis or resistance to therapy. This review summarizes the spectrum and frequency of co-mutations in NSCLC, their prognostic and predictive implications, and their relevance to targeted therapy and immune checkpoint inhibitors (ICIs). Understanding co-mutational profiles is critical to improving precision oncology strategies and tailoring treatment in advanced NSCLC.

## 1. Introduction

Non-small cell lung cancer (NSCLC) accounts for about 85% of lung cancers and is a leading cause of cancer mortality [1]. In recent years, molecular profiling has identified numerous oncogenic driver mutations (i.e., *EGFR*, *Epidermal Growth Factor Receptor*; *ALK*, *Anaplastic Lymphoma Kinase*; *KRAS*, *Kirsten Rat Sarcoma Viral Oncogene*; *ROS1*, *c-ros Oncogene 1*; *BRAF*, *B-Raf Proto-Oncogene*; *MET*, *MET Pro-to-Oncogene*; *RET*, *Ret Pro-to-Oncogene*; *NTRK*, *Neurotrophic Tyrosine Receptor Kinase*) which occur in roughly 40–50% of NSCLC cases [2]. These discoveries have revolutionized treatment through targeted therapies, substantially improving clinical outcomes. Tyrosine kinase inhibitors (TKIs) directed at *EGFR* mutations or *ALK/ROS1* rearrangements, for example, are now standard first-line treatments that have extended survival for many patients [3]. Precision medicine in NSCLC is predicated on the concept of a single dominant driver alteration guiding therapy; however, growing evidence shows this paradigm is overly simplistic. Tumors often harbor co-occurring genomic alterations alongside the primary driver, which can significantly influence tumor biology, prognosis, and treatment response [4].

**Figure 1:**
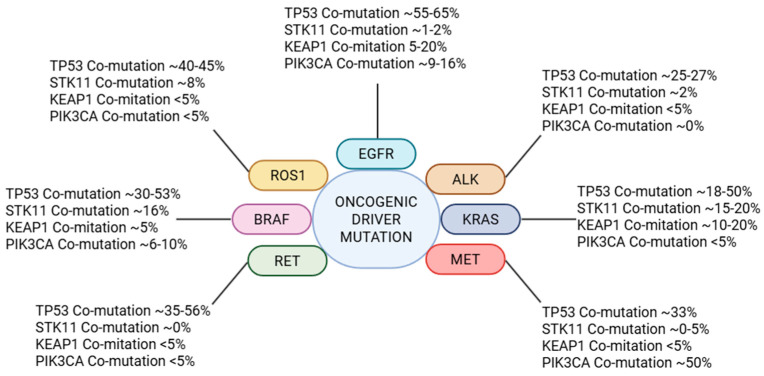
An overview of co-occurring genomic alterations in NSCLC. Abbreviations: ALK, Anaplastic Lymphoma Kinase; BRAF, B-Raf Proto-Oncogene; EGFR, Epidermal Growth Factor Receptor; KEAP1, Kelch-like ECH-associated protein 1; KRAS, Kirsten Rat Sarcoma Viral Oncogene; MET, MET Proto-Oncogene; PIK3CA, Phosphoinositide 3-kinase; RET, Ret Proto-Oncogene; ROS1, c-ros Oncogene 1; STK11, Serine/Threonine Kinase 11; TP53, Tumor Protein p53 (Created with www.BioRender.com, Licensing and Agreement number EJ28GM5B9V, accessed on 2 July 2025).

Co-mutations in NSCLC refer to additional pathogenic gene alterations (mutations, copy number changes, rearrangement) that exist in the same tumor as an established driver mutation or fusion. These may include loss-of-function mutations in tumor suppressors (i.e., *TP53*, *Tumor Protein p53; STK11*, *Serine/Threonine Kinase 11; KEAP1*, *Kelch-like ECH-associated protein 1*), activation of parallel signaling pathways (i.e., *PIK3CA*, *Phosphoinositide 3-kinase* mutations), or even a second actionable driver in rare cases, depicted in Figure 1. Such co-mutations contribute to intra-tumor molecular heterogeneity and can drive clonal evolution of the cancer [5,6,7]. Importantly, the presence of co-alterations has been linked to variability in clinical behavior—for instance, differences in growth rate, metastatic pattern, and drug sensitivity within what was thought to be a single molecular subtype of NSCLC [4].

In *EGFR*-*mutant* lung adenocarcinoma, for example, concurrent *TP53* mutation defines a subgroup with distinctly poorer responses to EGFR TKIs [8,9]. More broadly, oncogene-driven clusters are heterogeneous with respect to molecular profiles, clinical outcomes, and responsiveness to targeted therapies, and co-mutations are increasingly recognized as key contributors to this cluster heterogeneity.

From a clinical standpoint, it can have several implications. Molecularly, co-occurring genomic alterations may indicate a more complex tumor biology, with multiple pathways driving growth. Prognostically, certain co-mutations portend more aggressive disease or shorter survival. Predictively, co-mutations can modulate sensitivity or resistance to therapies—both targeted kinase inhibitors and immunotherapies.

In this review, we aim to provide a comprehensive overview of co-occurring genomic alterations in NSCLC, discussing the frequency and spectrum of major co-mutations across different molecular subtypes of NSCLC, their evidence for prognostic and therapeutic impacts, and how co-mutational landscapes can impact and be harnessed to overcome resistance.

## 2. Co-Occurrence of Genomic Driver Alterations Across NSCLC Subtypes

Extensive genomic profiling of NSCLC has revealed that most tumors with actionable drivers also harbor additional mutations. In a previous study from our group, involving 284 advanced NSCLCs with known driver alterations, 82.8% of cases had at least one co-occurring pathogenic variant or copy number alteration identified [5]. Similarly high rates of co-mutation have been reported in other cohorts [10,11].

These co-alterations range from single “passenger” mutations to multiple concurrent driver-level events. Table 1 summarizes the prevalence of several important co-mutations observed in NSCLC subsets defined by different primary drivers [5,10,12,13,14,15,16].

### 2.1. Frequency and Distribution of Main Co-Mutations by Driver Gene

*TP53* is by far the most common co-mutated gene across almost all oncogenic subgroups of NSCLC. More than half of *EGFR-mutant* lung adenocarcinomas have a concurrent *TP53* mutation [11], and TP53 co-mutations are also frequent in tumors with *ALK*, *ROS1*, and *RET* fusions (approximately 25–50%, Table 1), [14,15]. *KRAS-driven* NSCLC shows *TP53* mutation rates ranging up to 50% depending on patient demographics [12].

In contrast to *TP53*, *STK11 (LKB1)* mutations show a strong predilection for *KRAS-mutant* cancers. In total, 15–20% of *KRAS-mutant* tumors have *STK11* co-mutation, significantly higher than the rates reported in *EGFR* or *ALK* subgroups [5,14]. *KEAP1* mutations—which often co-occur with *STK11* in smoking-related lung cancers—are also enriched in *KRAS-mutant* NSCLC, found in roughly 10–20% of *KRAS* G12C tumors, defining a distinct subset [17,18].

Meanwhile, PIK3CA mutations or amplifications are recurrent as secondary events in certain subsets: about 10–15% of *EGFR-mutant* tumors have a PIK3CA pathway co-alteration, and ~10% of *BRAF-mutant* tumors as well [5]. Amplifications or gains in cell cycle genes (i.e., CCND1, CDK4; often measured as the “cycline-related” category including CDKN2A/B loss) are another frequent class of co-aberration, particularly in *MET exon14* skipping tumors (up to 50%) and *EGFR-mutants* (~20%), [5].

### 2.2. Driver Actionable Co-Alterations

Historically, driver mutations were thought to be mutually exclusive, but with more sensitive next-generation sequencing (NGS), this “rule” has been broken. Truly concurrent actionable driver alterations (i.e., two different drivers in one tumor) are rare but not negligible. In a large series of 1520 NSCLC patients, only 26 cases (1.7%) harbored compound actionable mutations (such as *EGFR* mutation plus a fusion), [19]. The most frequent combinations involved EGFR, with a concurrent *EGFR* mutation and *ROS1* fusion reported as the top association in the cohort [19]. Dual *EGFR+ALK*, *EGFR+RET*, *ALK+KRAS*, and even *BRAF+ALK* have also been documented, though each individual combination is exceedingly infrequent (often <1% incidence), [20,21]. Large studies report *EGFR–ALK* co-alterations in approximately 1–3% of cases when broad NGS panels are used [20,22].

Distinctive patterns of co-mutational genomic subtypes of NSCLC are probably related to the timing of the driver event during tumor evolution [23]. Gene fusion-driven tumors (*ALK*, *ROS1*, *RET*, etc.) tend to have overall fewer co-occurring mutations, implying the fusion arises early as a singular genomic hit. In contrast, *KRAS-*, *EGFR-*, *MET-*, or *BRAF-driven* tumors—especially in smokers—often exhibit a higher number of additional alterations. This dichotomy hints at different clonal evolution models [23,24]. Fusion-positive cancers may follow a more linear evolutionary trajectory, where the gene rearrangement is the dominant early event, and the tumor accumulates fewer other alterations over time.

On the other hand, tumors with point mutations like *KRAS* or *EGFR* may follow a branched evolution, developing multiple subclones in parallel, each acquiring diverse passenger or even additional driver mutations over the course of tumor development [23,24]. The co-existence of multiple subclonal populations in branched-evolution tumors can lead to competition and selection, particularly under treatment pressure, fostering drug-tolerant clones and eventual resistance.

### 2.3. Co-Occurring Epigenetic and DNA Repair Alterations in NSCLC

The occurrence of concurrent genomic alterations in genes involved in epigenetic regulation, DNA damage response (DDR), and chromatin remodeling has emerged as a critical determinant of tumor biology, therapeutic resistance, and clinical outcomes [25]. These alterations, which frequently co-exist with canonical driver mutations or alterations (such as *EGFR*, *KRAS*, or *ALK*), contribute to intratumoral heterogeneity and impact sensitivity to both targeted therapies and immune checkpoint inhibitors (ICIs), [26].

Among the most recurrent are inactivating mutations in chromatin remodelers such as *SMARCA4*, observed in 5–10% of NSCLC patients, particularly those with *KRAS-mutant* or smoking-associated tumors [27]. *Schoenfeld and colleagues* identified two clinically distinct classes of *SMARCA4* alterations in metastatic NSCLC, both significantly enriched in tumors co-mutated with *KRAS*, *STK11*, and *KEAP1*, compared to *SMARCA4 wild-type* (*p* < 0.001), [28]. Although both classes were associated with adverse clinical outcomes, Class 1 mutations, comprising truncating variants, gene fusions, and homozygous deletions, were characterized by loss of SMARCA4 protein expression and represented the most powerful independent negative prognostic factor. Notably, despite their unfavorable prognostic significance, patients with *SMARCA4-mutant* tumors, especially those harboring Class 1 alterations, derived greater clinical benefit from ICI therapy compared to those who did not receive ICI [28]. Multiple retrospective analyses have demonstrated that patients with dual mutations (i.e., *KRAS* plus *SMARCA4*) have significantly shorter PFS and OS when treated with first-line chemo-immunotherapy, compared to patients with only *KRAS* mutations [29]. *Dy GK and coworkers* reported a median PFS of 2.2 months and OS of 6.6 months in *KRAS+SMARCA4* co-mutated patients versus 6.2 and 14.6 months, respectively, in those with *KRAS* mutation alone [30].

Moreover, *SMARCA4-deficient* tumors have been shown to exhibit a dedifferentiated histology, aggressive clinical behavior, and resistance to multiple therapeutic modalities, including chemotherapy and immunotherapy. These tumors often display a suppressed interferon signaling signature and reduced immune infiltration, contributing to an immunologically “cold” tumor microenvironment [27,31]. In particular, *SMARCA4-deficient* NSCLC has been associated with lower levels of STING pathway activation and diminished recruitment of CD4^+^ and CD8^+^ T-cells, resulting in poor response to PD-1/PD-L1 blockade [32].

Furthermore, alterations in *DDR* genes such as *ATM* and *ATRX* also play a significant role in shaping tumor evolution and therapeutic outcomes [33]. Nonsynonymous *ATM* mutations occur in approximately 11% of NSCLC samples and frequently co-occur with *KRAS* mutations, while showing mutual exclusivity with *EGFR*, *TP53*, and *KEAP1* within the *KRAS*-mutant subset from clinicogenomic database (CGDB). Co-mutations with *TP53* are often missense and linked to high TMB, and potential increased responsiveness to ICI when combined with DNA-damaging chemotherapy [34]. This can paradoxically sensitize tumors to poly (ADP-ribose) polymerase (PARP) inhibitors, although responses are often context-dependent and influenced by additional co-mutations [35].

*ATRX*, involved in chromatin remodeling and telomere maintenance, is mutated in a smaller subset of NSCLC but has been implicated in resistance to immunotherapy via impaired antigen presentation and immune evasion [36]. These mutations are frequently associated with a higher burden of gene mutations (particularly *TP53*) and copy number variations (i.e., *CDKN2A*, *CDKN2B).* Although patients with *ATRX-mutated* tumors exhibit slightly improved OS and PFS, these differences are not statistically significant. In *ATRX-deficient* Lewis lung cancer cell line (LLC-sgAtrx), this deficiency enhances sensitivity of lung cancer cells to ICIs through multiple mechanisms [37].

From a therapeutic standpoint, these findings underscore the need for novel combination strategies. Current trials are exploring the use of epigenetic modulators—such as Enhancer of Zeste Homolog 2 (EZH2) inhibitors, histone deacetylase (HDAC) inhibitors, and bromodomain and extraterminal domain (BET) inhibitors—in combination with ICIs or DDR-targeted therapies (i.e., ATR, Checkpoint Kinase 1, CHK1; or PARP inhibitors) to overcome resistance in tumors harboring such co-alterations [38]. For instance, the SWI/SNF deficiency caused by *SMARCA4* loss has been shown to increase dependency on EZH2-mediated repression, providing a potential synthetic lethality approach [39]. Additionally, integrating comprehensive genomic profiling through NGS, including assessment of epigenetic and *DDR* co-alterations, is becoming essential for personalized risk stratification and guiding treatment in advanced NSCLC [40].

## 3. Prognostic Impact and Therapeutic Outcomes of Co-Occurring Genomic Alterations

As previously discussed, co-mutations can substantially affect prognosis and treatment outcomes in oncogene-driven NSCLC. In general, the presence of certain co-mutations is associated with more aggressive tumor behavior and shorter survival, as well as diminished efficacy of targeted therapies [18]. On the other hand, understanding co-mutation patterns also opens opportunities for tailoring treatment (for example, adding or prioritizing certain therapies).

### 3.1. EGFR-Mutant NSCLC

*EGFR-mutant* lung adenocarcinoma is a prime example where co-mutations stratify patient outcomes. The tumor suppressor *TP53* is co-mutated in approximately 50–65% of EGFR-positive tumors, making it the most common concurrent alteration in this subgroup [4,5]. Mounting clinical data indicates that *EGFR-mutant* tumors with *TP53* co-mutations are far worse than those with *wild-type TP53* [9]. A 2023 meta-analysis encompassing 29 trials found that among advanced *EGFR-mutant* NSCLC patients treated with EGFR TKIs, those whose tumors harbored concurrent *TP53* mutations had significantly shorter progression-free survival (PFS) and overall survival (OS) compared to those with only the *EGFR* driver [41]. Specifically, *TP53 co-mutant* patients had about a 1.67-fold higher hazard of progression and 1.89-fold higher hazard of death versus *TP53 wild-type* counterparts [25]. These results were consistent regardless of whether first/second-generation or third-generation EGFR TKIs were used, confirming *TP53* as a negative prognostic factor in this setting. Recently, comprehensive genomic profiling by NGS was performed in a subset of patients (n = 51) in order to investigate therapeutic outcomes of NSCLC patients harboring uncommon *EGFR* mutations other than exon 20 insertions (20ins) and T790M who received first-line therapy of gefitinib/erlotinib/icotinib and afatinib. Patients without co-mutations had a significantly longer mPFS (31.1 months) compared to those with tumor-suppressor gene alterations (9.2 months) or co-occurring driver oncogenes (12.4 months). *TP53* emerged as the most frequent co-mutation and was strongly associated with shorter mPFS (7.0 vs. 31.1 months, *p* < 0.001). Finally, multivariate analysis confirmed that concurrent *EGFR* 19del/L858R predicted favorable prognosis, whereas tumor-suppressor gene alterations, particularly *TP53*, were independent negative prognostic markers [42].

This has implications for treatment because such patients might benefit from more aggressive, or combination approaches rather than EGFR TKI alone. Of note, very recently reported results from phase 3 combination trials in the front-line setting (FLAURA2 [43] and MARIPOSA [44]), show that upfront combinatorial approaches can improve survival outcomes compared to single-agent osimertinib also in the poor-prognostic subgroups, including *TP53* co-occurring mutations [45].

Beyond *TP53*, research is ongoing into other co-mutations that may influence EGFR-TKI outcomes. These findings all reinforce that the *EGFR* mutation alone does not fully determine prognosis and the co-mutational context matters [4].

Other co-mutations in *EGFR*-*driven* NSCLC are less common but can also influence outcomes. *Retinoblastoma 1 (RB1)* alterations, when present with *EGFR*, have been associated with transformation into small-cell lung cancer under drug pressure, as a mechanism of acquired resistance [46]. Some *EGFR-mutant* tumors also harbor low-frequency co-alterations in the PI3K pathway (i.e., *PIK3CA*~10%); these may confer partial resistance to EGFR TKIs by activating parallel survival pathways [5].

Notably, *EGFR*-positive tumors generally respond poorly to immune checkpoint inhibitors [47]. Co-mutations might modulate this. Indeed, EGFR-mutant tumors that also carry *TP53* mutations (and often have higher tumor mutational burden, TMB) could be slightly more responsive to immunotherapy than *EGFR-mutant/TP53-wild-type* tumors, according to some reports [48]. Nevertheless, immunotherapy remains a secondary consideration in this population due to overall low efficacy [47].

### 3.2. KRAS-Mutant NSCLC

*KRAS* mutations represent the single most common driver in NSCLC (occurring in ~30% of adenocarcinomas, with the *KRAS G12C* variant comprising about 13% of cases), [12]. *KRAS-mutant* lung cancers display a particularly broad co-mutation profile, reflecting their frequent origin in smokers with attendant mutational damage. The three most frequently co-mutated genes in *KRAS-driven* NSCLC are *TP53*, *STK11*, and *KEAP1* [18]. Large real-world datasets show *TP53* co-mutations in ~18–50% of *KRAS G12C* tumors, *STK11* in ~10–28%, and *KEAP1* in ~6–23% [12]. These co-mutations define distinct phenotypic subsets with different outcomes.

Patients with *KRAS-mutant* lung adenocarcinoma and co-mutations in *STK11* and/or *KEAP1* generally have the poorest prognosis. *STK11 (LKB1)* loss and *KEAP1* loss both confer a more aggressive, therapy-resistant tumor biology. Clinically, *KRAS-mutant* tumors with *STK11/KEAP1* inactivation are known to be less responsive to immune checkpoint inhibitors and often exhibit a “cold” tumor immune microenvironment (low PD-L1, Programmed Death-Ligand 1, with low T-cell infiltration), [13].

A large-scale retrospective study, conducted by *Zhang F and collaborators*, involving 1745 patients across eight independent NSCLC cohorts systematically characterized the interaction effects of co-occurring mutations on the efficacy of ICI. In nonsquamous NSCLC, *KRAS/TP53* co-mutations were associated with improved PFS, whereas *KRAS/STK11* co-alterations conferred worse outcomes. Additional significant interaction was identified between *KRAS* and *Protein Tyrosine Phosphatase Receptor Type D (PTPRD)*, *RNA-Binding Motif Protein 10 (RBM10),* and *Neurogenic locus notch homolog protein* (*NOTCH1/2/3)*, reflecting novel immune-related mechanisms in *KRAS-mutant* lung adenocarcinomas. In squamous NSCLC, a significant synergistic effect was observed for *TP53/NFE2L2* co-mutations, potentially indicating enhanced immunogenicity [13].

From an immune-related perspective, most prominently TMB and the tumor immune microenvironment (TIME), play a pivotal role in modulating responses to ICIs, yet their integration with co-mutation status remains incompletely elucidated [49].

The well-known impact of KRAS signaling on the tumor microenvironment (TME) and immune response has raised high expectations for ongoing combination trials with ICI. Notably, the prevalence of high TMB (≥10 mutations/Mb) differs among *KRAS* mutation subtypes, correlating with an increased neoantigen load that enhances tumor immunogenicity in NSCLC [50,51].

High TMB correlates with increased neoantigen load, enhancing tumor immunogenicity and generally predicting better ICI responses in NSCLC [50,51]. However, *Biton J and collaborators* reported that the presence of co-occurring mutations in *STK11/LKB1* may counteract this benefit by promoting immune evasion through metabolic reprogramming, impaired antigen presentation, and recruitment of immunosuppressive cells such as myeloid-derived suppressor cells (MDCS) and regulatory T-cells [52]. These genomic-immune interactions yield distinct TIME phenotypes with important therapeutic implications. In this context, comprehensive immune profiling has demonstrated that NSCLC tumors harboring *KEAP1/STK11* mutations exhibit low PD-L1 expression, reduced interferon gamma signaling, and diminished infiltration of CD8+ T-cells, correlating with resistance to anti-PD-1/PD-L1 therapy despite high TMB [53,54]. Unlike tumors harboring somatic *TP53* mutations, KL tumors, which carry co-mutations in *KRAS* and *LKB1/STK11*, tend to be “immune-inert,” characterized by a lower infiltration of cytotoxic CD8+ T-cells. In contrast, KP tumors, defined by co-mutations in *KRAS* and *TP53*, display higher immunogenicity, with increased neoantigen burden and greater immune cell infiltration. These distinctions reported by *Scheffler M and colleagues* suggested that KP tumors may respond favorably to immune checkpoint inhibitors targeting the PD-1/PD-L1 axis, whereas KL tumors, due to their relatively “cold” immune microenvironment, might benefit from alternative therapeutic approaches [55].

The advent of direct KRAS G12C inhibitors has provided new targeted options (sotorasib and adagrasib approved in advanced NSCLC). Even here, co-mutations have prognostic value. A recent analysis from the KRYSTAL-1 trial (adagrasib in *KRAS G12C*-*mutant* NSCLC) showed that co-occurring *KEAP1* or *STK11* mutations were associated with significantly worse outcomes on KRAS G12C inhibitor therapy [56]. Patients with *KEAP1-mutant* tumors had a median PFS of only 4.1 months on adagrasib, compared to 9.9 months in *KEAP1-wild-type* patients [56]. Similarly, *STK11-mutant* patients had median PFS 4.2 vs. 11.0 months relative to *STK11-wild-type*. Conversely, patients whose tumors were *wild-type* for both *KEAP1* and *STK11* achieved markedly longer benefit (median PFS ~16.9 months), [56]. Preclinically, loss of *KEAP1* (which leads to activation of NRF2) has been shown to drive drug resistance by enabling tumor cells to withstand therapeutic stress; consistently, inhibition of KEAP1/NRF2 signaling or adding mTOR inhibitors showed promise in overcoming resistance in *KEAP1/STK11 co-mutant KRAS* models [56,57].

Interestingly, not all co-mutations in *KRAS* patients portend poor outcomes. The presence of *TP53* co-mutation in *KRAS-mutant* NSCLC has been associated with an inflamed phenotype and better responses to immunotherapy. In nonsquamous NSCLC, *KRAS* and *TP53* frequently co-occur, which correlates with higher PD-L1 expression and TMB [13]. Thus, co-mutations can have opposite predictive implications depending on which genes are involved—*TP53* co-mutation may predict better immunotherapy response, while *STK11* or *KEAP1* predict primary resistance.

With the introduction of *KRAS*-targeted treatments, retrospective efforts have also examined broad genomic factors influencing outcomes. A 2023 multi-center study of 424 patients treated with sotorasib or adagrasib identified *KEAP1*, *SMARCA4* (*SWI/SNF-Related*, *Matrix-Associated*, *Actin-Dependent Regulator of Chromatin*, *Subfamily A*, *Member 4*) and *CDKN2A* (*Cyclin-Dependent Kinase Inhibitor 2A*) mutations as significantly associated with early progression on therapy [58]. Mutations in each of those three genes were independently linked to shorter PFS/OS, whereas mutations in *STK11* or *TP53* alone did not show a significant association in that analysis. About one-third of *KRAS G12C* patients had one of (or a combination of) *KEAP1*, *SMARCA4*, *CDKN2A* alterations, and these accounted for roughly half of the cases of rapid progression (within 3 months) on KRAS G12C inhibitor monotherapy. This suggests that, beyond the well-known *STK11/KEAP1*, alterations in other tumor suppressors like *SMARCA4* (which often co-mutates with *KRAS* in smokers) and cell cycle regulators (*CDKN2A* loss) can also diminish the efficacy of KRAS inhibitors. Exploratory analysis in that study hinted those co-mutations in DNA damage repair genes (i.e., *ATM*, *Ataxia-Telangiectasia Mutated; ATRX/DAXX*, *Alpha Thalassemia/Mental Retardation Syndrome X-linked/Death-Domain-Associated Protein* pathway) were associated with improved outcomes on KRAS G12C inhibitors [58]—possibly because they confer a more genomically unstable phenotype that is more sensitive to therapy. These observations, while needing prospective validation, illustrate how the broader co-mutation context can modulate drug response in *KRAS-driven* NSCLC.

### 3.3. Other Oncogenic Drivers and Compound Co-Mutations

For NSCLC driven by *ALK*, *ROS1*, or *RET* fusions or by less common mutations (*MET* exon 14 skipping, *BRAF V600E*, *HER2- Human Epidermal Growth Factor Receptor 2- exon20* insertions, etc.), co-occurring alterations also play a role, though data are somewhat less extensive than for *EGFR* or *KRAS*. Generally, the presence of *TP53* mutations emerges as a recurrent theme in many of these subtypes, often correlating with worse outcomes on targeted therapy.

In *ALK*-rearranged NSCLC, the impact of *TP53* mutation is significant: a recent real-world analysis (GuardantINFORM database) of ALK TKI outcomes found that detection of a *TP53* mutation was associated with a hazard ratio of 1.53 for earlier discontinuation of first-line ALK TKI therapy [59]. Median time to drug discontinuation was about 17.3 months with *TP53-mutant* vs. not reached in *TP53 wild-type ALK* patients. Furthermore, *ALK+* tumors with *TP53* mutation have been linked to a higher risk of developing brain metastases on ALK TKI and a shorter duration of disease control [59]. The GuardantINFORM study noted that having the *ALK* fusion variant 3 (v3) and a *TP53* mutation together was particularly deleterious, with a median TKI duration of only 7.4 months in that subgroup. In contrast, patients with *ALK* variant 1 and no *TP53* mutation had much longer benefit (~20–32 months median before discontinuation), [59].

For *ROS1*- and *RET*-rearranged NSCLC, specific outcome data are lacking due to smaller numbers, and whether *TP53* affects the efficacy of targeted inhibitors (such as selpercatinib, pralsetinib) is still under investigation, but some early analyses indicate *TP53-mutant* RET+ patients have numerically shorter PFS on these drugs [60,61]. Similarly, prognostic or predictive role of co-occurring alterations in other driver mutations, besides their incidence, are based on anecdotal evidence.

Finally, the rare scenario of compound actionable mutations, where two driver alterations exist in the same tumor, deserve mention in terms of therapeutic outcome. Retrospective data suggest that outcomes are generally poorer than in single-driver disease, likely because the tumor behaves aggressively and is harder to target with a single agent [19]. In one series of 26 patients with concurrent actionable mutations, the median PFS on front-line targeted therapy was only 6 months– markedly shorter than typical PFS for a single driver [19].

This suggests an inherent biologic aggressiveness—possibly due to parallel dominant clones or higher genomic instability. As a result, compound driver cases often warrant chemotherapy-based treatment.

## 4. Acquired Resistance Mechanisms in Oncogenic Drivers of NSCLC

Under the selective pressure of targeted therapies, NSCLC tumors often acquire new genomic alterations that confer drug resistance. These acquired resistance mechanisms can be viewed as treatment-induced co-mutations that emerge over time. Understanding these mechanisms is crucial for developing strategies to overcome resistance and improve long-term outcomes, as outlined in Table 2 summarizing acquired resistance mechanisms and potential therapeutic strategies. The spectrum of resistance mechanisms varies by driver, but there are some common patterns: (1) secondary mutations in the target oncoprotein that prevent drug binding, (2) activation of bypass signaling pathways or alternate drivers, and (3) phenotypic/histologic transformation that alters drug sensitivity [6,46].

Importantly, the co-occurring genomic landscape prior to treatment can foreshadow some resistance mechanisms. For example, an *EGFR-mutant* tumor that also harbors a low-frequency *TP53* mutation at baseline might rapidly become resistant to EGFR TKI by outgrowth of the *TP53*-*driven* clone. Likewise, a minor *KRAS-mutant* subclone present before ALK inhibitor treatment could expand and cause early resistance (a scenario of primary resistance due to a co-mutation). This underscores the interplay between baseline co-mutations and acquired mutations: sometimes what we call acquired is really the selection of a pre-existing *co-mutant* clone.

Comprehensive upfront sequencing can occasionally detect these low-frequency co-drivers, which may prompt a different initial treatment approach (for instance, combination therapy).

To combat acquired resistance, several strategies are being pursued. One is the development of next-generation inhibitors that can target the resistant mutants [62]. Another approach is combination therapy: adding an MET inhibitor to overcome *MET*-*driven* resistance to EGFR TKIs, or combining SHP2 (Src Homology 2 Domain-Containing Phosphatase 2) or MEK (Mitogen-Activated Protein Kinase) inhibitors with KRAS G12C inhibitors to suppress bypass tracks [63,64]. Similarly, combining a CDK4/6 (Cyclin-Dependent Kinase 4/6) inhibitor might help in cases where *CDKN2A* loss drives resistance [46].

There is also interest in sequencing and switching therapies at appropriate times (for instance, using chemotherapy or immunotherapy intercalated with targeted therapy to eliminate heterogenous clones), [6]. A key practical strategy is re-biopsy at progression: performing NGS on tumor tissue or plasma at the time of acquired resistance to identify the new mutations [19].

Notably, tumors with rich co-mutation profiles tend to have multiple resistance mechanisms concurrently (inter- and intra-tumor heterogeneity of resistance), [24,65]. For example, different metastatic lesions in an *EGFR-mutant/TP53-mutant* patient might show different resistance mutations [66]. This heterogeneity makes overcoming resistance challenging, because treating one mechanism may not address another. Such cases often require combination treatments or systemic therapies that cover all bases (i.e., chemotherapy can non-specifically kill cells regardless of the precise resistance mutation).

## 5. The Role of Liquid Biopsy in Evaluating Co-Mutations in Lung Cancer

In the era of high-throughput molecular profiling and personalized medicine, determining the true “actionability” of cancer mutations in NSCLC remains a challenging and unresolved issue [67,68]. Blood-based liquid biopsies, including circulating tumor cells (CTCs), circulating tumor DNA (ctDNA), and exosomes, offer a rapid, non-invasive approach to capture tumor genetic heterogeneity and temporal genomic evolution [69,70]. Next-generation sequencing (NGS) of ctDNA enables comprehensive molecular profiling by identifying actionable drivers extended to somatic mutations (i.e., *EGFR*, *ALK*, *ROS1*), resistance-associated mutations (i.e., *EGFR T790M*), and co-occurring genomic alterations, thereby supporting early cancer detection, patient stratification, dynamic monitoring of disease progression, and evaluation of treatment response to guide personalized therapeutic decisions [71].

Table 3 provides an overview of the most relevant co-occurring genomic alterations in NSCLC, outlining their functional roles, clinical implications, mechanisms of therapeutic resistance, and the utility of ctDNA-based liquid biopsy for their detection and monitoring.

*Chabon and colleagues* profiled ctDNA in 100 early-stage NSCLC patients, achieving over 85% sensitivity in detecting key mutations and demonstrating reduced immunotherapy responses in patients with *KEAP1/STK11* co-mutations [72]. Similarly, ctDNA-detected *KEAP1* and *STK11* co-mutations in advanced NSCLC were found to be associated with a lower objective response rate (12% vs. 38%, *p* = 0.01) and shorter median PFS (2.8 vs. 6.7 months, *p* = 0.005) compared to *wild-type* patients [73].

Beyond targeted therapies, ctDNA profiling offers vital insights for immunotherapy decisions. For instance, longitudinal ctDNA tracking can reveal minimal residual disease (MRD) and relapse months before radiographic evidence [74]. The TRACERx study highlighted ctDNA dynamics post-surgery as an early predictor of recurrence [75,76]. In the B-F1RST trial (NCT02848651), the potential of blood-based tumor mutational burden (bTMB), assessed using the FoundationOne Liquid CDx assay, was prospectively evaluated as a predictive biomarker for first-line atezolizumab in advanced NSCLC patients unselected for PD-L1 status. Using a predefined cutoff of ≥16 mutations/Mb, patients with ctDNA detectable at a maximum somatic allele frequency ≥1% and high bTMB showed improved ORR and longer PFS [77].

The MYSTIC trial revealed that NSCLC patients exhibiting high plasma bTMB (≥20 mutations/Mb) achieved better OS, PFS, and ORR when treated with dual checkpoint blockade (durvalumab plus tremelimumab) compared to chemotherapy [78]. Technical advances in digital droplet PCR (ddPCR) and ultra-sensitive NGS platforms have enhanced ctDNA detection of frequent and rare alterations, making liquid biopsy a promising tool for NSCLC management, though further refinement and trials are needed for routine use [79,80].

### Complementary Role of Tissue and Liquid Biopsy NGS in Detecting Co-Occurring Mutations and Guiding NSCLC Treatment

A growing body of evidence underscores the complementary utility of tissue-based and plasma-based NGS for comprehensive genomic profiling in NSCLC [81]. Comparative analyses demonstrate that while FFPE tissue remains the gold standard for detecting somatic mutations, with variant allele frequency (VAF) thresholds typically ≥5%, ctDNA from liquid biopsy provides unique insights by capturing intratumoral and spatial heterogeneity not always evident in a single biopsy [82].

In a real-world, multicenter study of 232 advanced NSCLC patients across 11 Italian institutions, comprehensive genomic profiling was conducted using FoundationOne (F1CDx) and FoundationLiquid CDx (F1L/F1LCDx) NGS assays [83]. Significant differences were observed between tissue-based and ctDNA NGS, particularly *KRAS* mutations that were notably more frequent in samples analyzed with the tissue-based F1CDx assay compared to those assessed by ctDNA-based panels (F1L/F1LCDx). The actionability rates for tier I (variants with strong clinical evidence) and II (variants with potential clinical significance) alterations were similar between platforms, with 36.2% detected by F1CDx and 34% by ctDNA-based NGS assays (29.5% in F1L and 40.9% in F1LCDx). Notably, *KEAP1* mutations showed significant co-occurrence with *STK11* and *KRAS* alterations, while *TP53* mutations were commonly linked to *RB1* changes [83].

In a separate Ion Torrent-based study analyzing 41 cfDNA samples, *KRAS* mutations were found in 43.9% of patients, *TP53* in 31.7%, and *PIK3CA* in 29.3%, with significant co-occurrence of *KRAS+TP53* (14.6%) and *KRAS+PIK3CA* (17.1%). Notably, *TP53* mutation and high cfDNA levels independently predicted worse progression-free survival (hazard ratios 2.49 and 3.43, respectively), [84].

The prognostic and predictive significance of common tumor-associated genetic alterations detected in plasma was evaluated before initiating immune ICI in advanced NSCLC patients starting from a total of 103 patients whom they were prospectively screened using plasma NGS within two trials—VISION (Guardant360^®^) and MAGIC (Myriapod NGS-IL 56G Assay), [85]. *TP53* mutations detected in plasma were associated with significantly shorter OS in both ICI-treated and control cohorts, supporting their prognostic value. *STK11* mutations showed a trend toward worse OS only in the ICI group. Importantly, *KRAS/STK11* and *KRAS/STK11/TP53* co-mutations were linked to significantly poorer OS exclusively in patients receiving ICIs (HR = 10.936 and 17.609, respectively), suggesting a predictive role in resistance to immunotherapy [85].

Recently, a comparative Oncomine NGS analysis from both tissue and plasma in NSCLC patients revealed that 24.13% (7/29) of mutations—such as *EGFR L858R*, *KRAS G13D/Q61H*, and several *TP53* variants—were detected exclusively in plasma cfDNA, not in the tumor tissue. These mutations, found in seven patients (two with distant metastases), highlight the ability of cfDNA-based NGS to uncover actionable alterations missed by tissue analysis in an ATLAS study from Spain [86].

These findings support the integrated use of tissue and liquid NGS to maximize detection sensitivity, monitor clonal evolution, and identify co-occurring alterations with prognostic or therapeutic significance in NSCLC. Such promising results highlight the growing importance of combining these approaches in clinical practice and warrant further investigation [87].

## 6. Conclusions

The growing catalog of co-occurring genomic alterations in NSCLC has important implications for research and clinical care. First, it is now clear that the presence of co-mutations can markedly influence prognosis and the effectiveness of therapy in oncogene-driven NSCLC. Integrating co-mutation analysis into routine practice could enable more personalized treatment.

A major challenge moving forward is the implementation of broad NGS testing for all advanced NSCLC patients to identify not just a single driver, but the full co-mutation profile. While guidelines increasingly recommend comprehensive genomic profiling, real-world adoption is uneven. Distinguishing which co-mutations are clinically relevant drivers versus incidental passengers will require continued research and possibly machine-learning approaches on big datasets. Multicenter collaborations will be necessary to correlate specific co-mutation constellations with clinical outcomes, given that single-institution cohorts may be underpowered for less common combinations.

From a treatment standpoint, one emerging approach is designing clinical trials specifically for co-mutant populations. Tailored therapeutic strategies based on co-mutation profiles are still mostly conceptual, but early-phase trials are starting to incorporate these ideas.

Another challenge is monitoring disease in the context of multiple co-clones. Liquid biopsy (circulating tumor DNA analysis) is emerging as a valuable tool to capture the full heterogeneity of co-mutations, especially at progression when tumors may be heterogeneous across sites [70,88]. Sensitive ctDNA assays can detect minor resistant subclones, potentially allowing preemptive therapeutic adjustments. However, interpreting complex ctDNA results (with numerous mutations) will require expertise and perhaps novel computational methods to deconvolute which combinations come from which tumor clone [89,90].

In conclusion, as we “unravel” the molecular and clinical co-alterations in NSCLC, we are learning that making order out of the crowded list of mutations is both challenging and necessary. Co-occurring genomic alterations contribute to tumor heterogeneity, impact prognosis (often negatively), and frequently drive both upfront therapy selection and mechanisms of resistance. Recognizing and addressing co-mutations is an emerging frontier in precision oncology. The final goal is to refine patient management such that a comprehensive genomic profile—encompassing both primary drivers and co-mutations—guides a truly personalized treatment plan for each NSCLC patient.

## 7. Future Directions

As the molecular landscape of NSCLC becomes increasingly complex with the identification of diverse co-occurring genomic alterations, traditional analytic methods are often insufficient to fully capture the intricate interactions driving tumor behavior and therapeutic response [91]. Machine learning (ML) and artificial intelligence (AI) offer powerful computational frameworks capable of integrating high-dimensional multi-omics data, as well as genomics, transcriptomics, and epigenomics, to uncover hidden patterns and predictive biomarkers associated with co-mutated NSCLC subtypes [92,93].

Recent studies demonstrate that ML algorithms can effectively stratify patients based on combined mutation profiles, improving prognostic accuracy and informing precision treatment selection [94]. Furthermore, ML approaches facilitate real-time interpretation of longitudinal liquid biopsy data, enabling dynamic monitoring of clonal evolution and early detection of resistance mechanisms [95,96]. Complementing computational advances, innovative clinical trial designs such as adaptive, umbrella, and basket trials are increasingly necessary to address therapeutic challenges posed by co-mutated NSCLC populations [97].

These designs allow flexible patient stratification based on molecular characteristics and permit the evaluation of targeted therapies or combination regimens within genetically defined subgroups, accelerating drug development and personalized therapy implementation [98,99]. For instance, the Lung-MAP trial employs a biomarker-driven umbrella design to test multiple targeted agents in NSCLC patients stratified by genomic alterations, including co-mutations [100]. Such trials enable more efficient assessment of treatment efficacy and resistance patterns in heterogeneous co-mutated cohorts, ultimately improving clinical outcomes.

Together, the integration of ML-driven data analysis and innovative clinical trial designs represents a promising frontier in overcoming the complexity of co-mutations in NSCLC, paving the way for more effective personalized therapeutic strategies [101].

## Figures and Tables

**Table 1:** Prevalence of selected co-mutations in NSCLC tumors harboring common driver alterations [5,10,12,13,14,15,16].

Primary Driver Alteration	*TP53* Co-Mutation (%)	*STK11* Co-Mutation (%)	*KEAP1* Co-Mutation (%)	*PIK3CA* Pathway Co-Mutation (%)
***EGFR*** mutation	~55–65%	~1–2%	5–20%	~9–16%
***KRAS*** mutation	18–50%	~15–20%	~10–20%	<5%
***ALK*** fusion	~25–27%	~2%	<5%	~0%
***ROS1*** fusion	~40–45%	~8%	<5%	<5%
***RET*** fusion	~35–56%	~0%	<5%	<5%
***MET*** exon 14 skipping	~33%	~0–5%	<5%	~50%
***BRAF*** mutation (V600/non-V600)	~30–53%	~16%	~5%	~6–10%

*Note*: Ranges reflect variability across studies; co-mutation percentages are approximate. PIK3CA pathway includes mutations in *PIK3CA* or related PI3K/AKT signaling genes. Co-mutations listed are not exhaustive; other alterations (i.e., *CDKN2A/B* deletions, *RB1* mutations, etc.) also occur but are not shown for brevity. Abbreviations: EGFR, Epidermal Growth Factor Receptor; KRAS, Kirsten Rat Sarcoma Viral Oncogene; ALK, Anaplastic Lymphoma Kinase; ROS1, c-ros Oncogene 1; RET, Ret Proto-Oncogene; MET, MET Proto-Oncogene; BRAF, B-Raf Proto-Oncogene; TP53, Tumor Protein p53; STK11, Serine/Threonine Kinase 11; KEAP1, Kelch-like ECH-associated protein 1; PIK3CA, Phosphoinositide 3-kinase.

**Table 2:** Summary of acquired resistance mechanisms in oncogenic driver-positive NSCLC patients and their implications for overcoming resistance.

Mechanism of Acquired Resistance	Examples
**Secondary mutations in the target gene**	*EGFR T790M* mutation contributes to resistance to first-generation EGFR TKIs
**Activation of alternative signaling pathways**	*MET* amplification can result in resistance to EGFR TKIs; *KRAS* subclonal expansion may lead to resistance to ALK TKI
**Phenotypic transformation**	Small cell transformation of *EGFR-mutant* NSCLC following TKI treatment
**Pre-existing co-mutations**	*TP53* or *KRAS mutant* subclones can be able to expand under EGFR or ALK TKI therapy, contributing to resistance
**Multiple concurrent resistance mechanisms**	Patients with *EGFR/TP53* mutations show a heterogeneous resistance across several metastatic sites
**Strategies to overcome resistance**	
**Next-generation inhibitors**	Osimertinib is administered for *EGFR T790M*
**Combination therapies**	EGFR TKIs with MET inhibitors, KRAS inhibitors with SHP2/MEK inhibitors, and CDK4/6 inhibitors for *CDKN2A* loss are used to bypass resistance and improve treatment effectiveness
**Sequential and intercalated therapy**	Chemotherapy or immunotherapy, intercalated with TKIs, aims to prevent the dominance of resistant clone
**Re-biopsy and liquid biopsy at progression**	Performing NGS on tissue or plasma samples to identify new targetable alterations

Abbreviations: EGFR, Epidermal Growth Factor Receptor; T790M, Threonine 790 to Methionine mutation; TKIs, Tyrosine Kinase Inhibitors; MET, MET Proto-Oncogene; KRAS, Kirsten Rat Sarcoma viral oncogene homolog; ALK, Anaplastic Lymphoma Kinase; NSCLC, Non-Small Cell Lung Cancer; TP53, Tumor Protein p53; SHP2, SH2-containing Protein Tyrosine Phosphatase 2; MEK, Mitogen-Activated Protein Kinase; CDK4/6, Cyclin-Dependent Kinase 4/6; CDKN2A, Cyclin-Dependent Kinase Inhibitor 2A; NGS, Next-Generation Sequencing.

**Table 3:** Key co-occurring genomic alterations in NSCLC: functional role, clinical implications, and liquid biopsy utility.

Gene/ Pathway	Functional Role	Common Co-Mutation Context	Clinical Implications	Therapeutic Resistance	ctDNA/Liquid Biopsy Utility
***STK11 (LKB1)***	Tumor suppressor, metabolic regulation	*KRAS-mutant* adenocarcinoma	Poor prognosis, immune “cold” phenotype	Resistance to PD-1/PD-L1 checkpoint inhibitors	Detectable via ctDNA for early risk stratification and treatment selection
***KEAP1***	NRF2 inhibitor, redox homeostasis	*KRAS/STK11* co-mutations	Poor outcome, therapy resistance, oxidative stress response	Resistance to ICIs and chemotherapy	Monitored in ctDNA to guide ICI eligibility and potential combination therapy
***TP53***	Genome integrity, cell cycle arrest	*EGFR*, *KRAS*, *ALK* mutations	Mixed prognostic value; associated with genomic instability	May confer resistance to TKIs when co-mutated with *EGFR*	High frequency in ctDNA; useful for tracking clonal evolution and therapy response
***SMARCA4 (BRG1)***	Chromatin remodeling, transcription regulation	Often with *KRAS* or *TP53* mutations	Associated with aggressive histology (i.e., large cell carcinoma), poor prognosis	Reduced response to both ICIs and chemotherapy	Detected in ctDNA; associated with dedifferentiated, aggressive phenotypes
***ATM/ATRX***	DNA damage response (DDR)	Co-mutated with *TP53*	Associated with genomic instability, potential ICI sensitization	Variable; may confer platinum sensitivity or ICI response	*DDR* mutations measurable in plasma; may inform on PARP inhibitor sensitivity
***MET*** **Amplification**	RTK signaling, bypass pathway	Acquired with EGFR TKI resistance	Mechanism of resistance to EGFR inhibitors	Resistance to osimertinib and earlier-gen EGFR TKIs	ctDNA enables detection of *MET* amplification; critical for initiating MET inhibitor therapy

Abbreviations: NSCLC, Non-Small Cell Lung Cancer; STK11 (LKB1), Serine/Threonine Kinase 11 (Liver Kinase B1); KRAS, Kirsten Rat Sarcoma viral oncogene homolog; PD-1, Programmed Cell Death Protein 1; PD-L1, Programmed Death-Ligand 1; ICI, Immune Checkpoint Inhibitor; ctDNA, Circulating Tumor DNA; KEAP1, Kelch-like ECH-associated protein 1; NRF2, Nuclear Factor Erythroid 2-Related Factor 2; TP53, Tumor Protein p53; EGFR, Epidermal Growth Factor Receptor; TKI, Tyrosine Kinase Inhibitor; SMARCA4 (BRG1), SWI/SNF-Related, Matrix-Associated, Actin-Dependent Regulator of Chromatin, Subfamily A, Member 4; ATM, Ataxia Telangiectasia Mutated; ATRX, Alpha Thalassemia/Mental Retardation Syndrome X-Linked; DDR, DNA Damage Response; RTK, Receptor Tyrosine Kinase; MET, Mesenchymal–Epithelial Transition factor.

## Data Availability

Not applicable.
